# The interplay between non-coding RNAs and Wnt/ß-catenin signaling pathway in urinary tract cancers: from tumorigenesis to metastasis

**DOI:** 10.17179/excli2022-5348

**Published:** 2022-10-18

**Authors:** Farzad Rahmani, Pegah Safavi, Ayda Fathollahpour, Fatemeh Tanhaye Kalate Sabz, Parastoo Tajzadeh, Mohsen Arefnezhad, Gordon A. Ferns, Seyed Mahdi Hassanian, Amir Avan

**Affiliations:** 1 Kashmar School of Nursing, Mashhad University of Medical Sciences, Mashhad, Iran; 2 Metabolic Syndrome Research Center, Mashhad University of Medical Sciences, Mashhad, Iran; 3 Basic Medical Sciences Institute, Mashhad University of Medical Sciences, Mashhad, Iran; 4 Department of Medical Radiation, Science and Research Branch, Islamic Azad University, Tehran, Iran; 5 Faculty of Medicine, Iran University of Medical Sciences, Tehran, Iran; 6 Brighton & Sussex Medical School, Division of Medical Education, Falmer, Brighton, Sussex, UK; 7 Medical Genetics Research Center, Mashhad University of Medical Sciences, Mashhad, Iran

**Keywords:** ncRNA, Wnt/beta-catenin, urinary tract cancers

## Abstract

Non-coding RNAs (ncRNAs) are emerging as important regulators in various pathological conditions including tumorigenesis, metastasis, and drug resistance in human cancers. Oncogenic or tumor suppressor ncRNAs exert prominent effects on cell proliferation, migration and invasion in cancer cells through modulating various signaling pathways including Wnt/β-catenin. Upregulation of the oncogenic Wnt/β-catenin pathway was reported to be implicated in multiple human cancers including breast, liver, colorectal, and urothelial cancers. Therefore, identifying interactions between ncRNAs and canonical Wnt signaling components may represent novel therapeutic targets for better treatment and management of cancer. In this review, we summarized the recent findings about miRNA/lncRNA-dependent mechanisms that regulate Wnt/β-catenin signaling involved in tumorigenesis and metastasis of urinary tract cancers.

## Introduction

Urinary tract (UT) cancers are the most common malignant tumors that can be located in different parts of the urinary system including the kidney, bladder, urethra, and prostate. Despite recent improvements in diagnostic and therapeutic methods, patients with UT cancers exhibit dismal prognosis and lower survival rates (Elumalai et al., 2021[11]; Yang et al., 2021[68]). Among all cancer types in the UT, bladder cancer showed the highest incidence and recurrence rate. UT patients are usually diagnosed in the advanced stages of cancer with poor prognosis and worse clinical outcomes (Powles et al., 2022[35]). Therefore, the identification of novel diagnostic and prognostic biomarkers may be useful for earlier diagnosis and effective treatment of UT patients. Recent molecular studies indicate that UT cancers arise from an accumulation of genetic mutations and epigenetic alterations that induce oncogenes or inhibit tumor suppressor genes resulting in upregulation of several oncogenic signalings including RAS, PI3K/ AKT, and Wnt/β-catenin signaling pathway (Sathe and Nawroth, 2018[43]; Kurtzeborn et al., 2019[26]; Chestnut et al., 2021[5]; Rahmani et al., 2021[40]; Wu et al., 2021[56]). Due to the critical function of the oncogenic signalings in regulating cancer cell proliferation, angiogenesis, and metastasis, aberrant activation of these pathways is considered as one of the major causes of human tumorigenesis (Soleimani et al., 2018[47]; Rahmani et al., 2020[41]). 

Wnt/β-catenin signaling (also known as the canonical Wnt pathway) plays critical effects on human carcinogenesis through inducing cell cycle progression, migration, invasion, and survival (Rahmani et al., 2018[39], 2019[38]). In canonical Wnt signaling, the cytoplasmic β-catenin protein has an essential role in signal transduction from frizzled transmembrane receptor to the nucleus and upregulation of Wnt signaling downstream genes. In the absence of the Wnt ligands, the canonical Wnt signaling is inactivated and cytoplasmic levels of β-catenin proteins are decreased by a destruction complex that induces phosphorylation, ubiquitination, and proteasomal degradation of β-catenin proteins (Yeh et al., 2019[68]). However, binding of Wnt ligand to the frizzled receptors at the surface of target cells triggers a chain of interactions which resulted in phosphorylation and inactivation of destruction complex proteins including AXIN-2, adenomatous polyposis coli (APC), glycogen synthase kinase 3β (GSK3β), and casein kinase1 (CK1). Following the inactivation of destruction complex, β-catenin proteins accumulated in the cytoplasm and were then imported into the nucleus. Subsequently, the nuclear β-catenin proteins interact with the certain regulatory transcription factors including T cell factor/lymphocyte enhancer factor (TCF/LEF) which are implicated in the transcription of various Wnt signaling downstream genes including cyclin D1, E-cadherin, and c-Myc (Sales et al., 2018[42]; He and Tang, 2020[16]; Zhang and Wang, 2020[77]).

Upregulation of Wnt ligands and β-catenin proteins, as well as decreased expression of the tumor-suppressors APC, AXIN-2, and GSK3β, were found to be associated with aggressive features in human cancers including brain, breast, hepatocellular (HCC), colorectal (CRC), and UT cancers (Krishnamurthy and Kurzrock, 2018[25]; Wang et al., 2019[53]; Yeh et al., 2019[68]; Shahcheraghi et al., 2020[44]; Zhang et al., 2021[71]). Emerging evidence indicates that ncRNAs have critical effects in regulating Wnt/β-catenin route which is upregulated in multiple cancers suggesting their significance as novel therapeutic options for cancer therapy (Lei et al., 2020[27]; Shao et al., 2020[45]; Takao Real Karia et al., 2021[50]). In the present study, we summarize the regulatory effects of ncRNAs on Wnt/β-catenin pathway in tumorigenesis and metastasis of UT cancers.

## Role of ncRNAs in Tumorigenesis

Previous findings in genome sequencing studies demonstrated that approximately 2 % of the human genome is transcribed to protein and the other RNA transcripts which do not encode proteins are considered as non-coding RNAs (Anastasiadou et al., 2018[1]). Generally, ncRNAs are classified into three main groups including miRNAs, circRNAs, and lncRNA based on shape and length. MiRNAs, identified as small RNAs with 20-25 nucleotides in length, were shown to have a potential regulatory effect on their target genes through interacting with the 3'-untranslated region of mRNA molecules (Bhan et al., 2017[2]). LncRNA molecules (longer than 200 nucleotides) with linear shapes play a prominent role in regulating target genes at transcriptional and post-transcriptional levels. Similarly, circRNAs are >200 nucleotides in molecular size with a circular structure that is resistant to the RNase enzymes. LncRNAs and circRNAs regulate gene expression by various mechanisms like sponging miRNAs to promote target genes. Recent studies indicate that dysregulated expression of ncRNAs promotes cancer growth and development by epigenetically regulating various oncogene or tumor suppressor genes (Chi et al., 2019[6]; Wei et al., 2020[55]; Jafarzadeh and Soltani, 2021[23]).

## The Interplay between ncRNAs and Wnt/β-catenin Pathway in Kidney Cancer

Renal cell carcinoma (RCC) as one of the most common types of urinary system malignancies is derived from the renal epithelium and accounts for more than 90 % of kidney cancers (Dahle et al., 2022[7]). Accumulating evidence revealed that GSK3β is downregulated in various tumors like breast, CRC, and RCC (Duda et al., 2020[9][10]). The tumor-suppressor GSK3β is shown to inhibit carcinogenesis by modulating the phosphorylation and degradation of β-catenin proteins and downregulation of Wnt signaling downstream target genes (Rahmani et al., 2020[41]). In RCC, the expression of GSK3β is suppressed by various lncRNAs including lung cancer-associated transcript 1 (LUCAT1) and colorectal neoplasia differentially expressed (CRNDE). Upregulation of the oncogenic lncRNA LUCAT1 (also known as SCAL1) is found to be correlated with lower survival rates and poor clinical outcomes in lung, gastric, HCC, CRC, and RCC patients (Wu et al., 2020[57]; Xing et al., 2021[62]). Increased expression of LUCAT1 induces tumor cell growth and development by regulating the AKT/GSK3β signaling pathway. Conversely, knockdown of LUCAT1 reduces cell cycle progression and inhibits tumor cell invasion and metastasis by downregulating cyclin D1 and β-catenin both *in vivo* and *in vitro* (Zheng et al., 2018[78]). 

LncRNA CRNDE is another oncogenic lncRNA that is upregulated in multiple tumors including glioma, gastric, CRC, and RCC (Xie et al., 2018[61]; Lu et al., 2020[30]). CRNDE located in chromosome 16q12.2 is actively implicated in tumorigenesis via induction of cell proliferation, invasion, and metastasis. Shao et al. reported that silencing CRNDE prevents cancer cell growth through promoting G1 phase cell cycle arrest. Further results demonstrated that the oncogenic activity of CRNDE is mediated by targeting GSK-3β resulting in activation of canonical Wnt pathway in RCC cells and tissues. These findings revealed that the GSK3β regulatory lncRNAs can be considered as novel therapeutic targets for better management of RCC (Yang et al., 2018[66]; Ding et al., 2020[8]; Zhang et al., 2021[75]).

To further explore the tumor-promotive role of ncRNAs, it has been shown that some lncRNAs can enhance RCC progression by directly promoting the expression of cyclin D1 and c-myc (Rahmani et al., 2018[39]). Leucine zipper-EF-hand containing transmembrane protein 1 (LETM1) as one of the cancer-related lncRNAs is shown to be upregulated in various human tumors including breast, bladder, and RCC (Huang et al., 2017[19]; Xu et al., 2018[63]). Aberrant expression of LETM1 was observed to be correlated with tumor cell metastasis and poor survival outcomes. It has been shown that Knockdown of LETM1 reduces the expression of β-catenin, cyclin D1, and c-myc in RCC cells (Xu et al., 2018[63]). Moreover, lncRNA colon cancer-associated transcript 2 (CCAT2) accelerates breast, prostate, glioma, and RCC proliferation and metastasis through inducing canonical Wnt pathway and promoting the expression of its downstream target genes (He et al., 2020[15]). Silencing of CCAT2 promotes a G1 phase cell cycle block and cell apoptosis suggesting its potential as a novel therapeutic target for RCC therapy (Huang et al., 2017[21]).

There are multiple lncRNAs that induce cancer development and drug resistance by sponging certain tumor-suppressive miRNAs and preventing their regulatory effects on downstream target genes. For instance, lncRNA HIF1A-AS2 accelerates tumor growth and metastasis through targeting miR-30a-5p (Chen et al., 2021[3]). Tumor suppressor miR-30a-5p was found to reduce tumor growth by repressing the expression of transcription factor SOX4 (Quan et al., 2019[37]). Recent findings uncovered that SOX4 has critical effects on epigenetically regulation of multiple genes involved in tumorigenesis of acute myeloid leukemia (AML), gastric cancer, and osteosarcoma (Ying et al., 2020[69]). Interestingly, downregulation of HIF1A-AS2 elicits growth-suppressive effects in RCC cells by downregulating cyclin D1, MET, c-myc, and VEGF. Therefore, these findings suggest that upregulation of lncRNA HIF1A-AS2 facilitates RCC growth through sponging miR-30a-5p and stimulating SOX4 which induces tumor cell growth and metastasis (Chen et al. 2021[3]).

Among all lncRNAs which are related to human cancers, downregulation of multiple lncRNAs was reported to be associated with RCC initiation and progression. For instance, decreased expression of LINC01510 was shown to be related to a poor prognosis in RCC patients. Ectopic expression of LINC01510 attenuates tumor growth and invasion by inhibiting β-catenin and various matrix metalloproteinases (MMPs) including MMP2, MMP7, and MMP9 (Ma et al., 2018[31]). In addition to LINC01510, downregulation of lncRNA neuroblastoma-associated transcript 1 (NBAT1) was reported in patients with RCC which is negatively associated with tumor growth and aggressiveness. Functionally, lncRNA NBAT1 functions as a ceRNA for miR-346 to upregulate GSK3β and mitigate cancer cell proliferation, invasion, and metastasis (Xue et al., 2019[64]). To further investigate the interplay between lncRNAs and miRNAs involved in RCC tumorigenesis, Zhang et al. (2020[73]) reported that the long intergenic ncRNA 1939 (LINC01939) regulates tumor cell growth and progression through downregulating miR-154. The overexpression of LINC01939 inactivates Wnt/β-catenin pathway through suppressing miR-154 in RCC. Mechanically, LINC01939 attenuates cancer cell growth and metastasis by downregulating cyclin D1, MMP2, and MMP9. Moreover, LINC01939 was found to induce RCC cell apoptosis through inducing the expression of pro-apoptotic factors including bax and caspase-3 (Zhang et al., 2020[73]).

The lncRNA OTUD6B antisense RNA 1 (OTUD6B-AS1) is another tumor-suppressive lncRNA that is downregulated in RCC patients and associated with lower survival rate and worse clinical outcomes. Ectopic expression of OTUD6B-AS1 inhibits tumor cell migration and invasion through suppressing the Wnt/β-catenin axis and reducing the expression of epithelial-to-mesenchymal transition (EMT)-related proteins including E-cadherin, N-cadherin, and Snail (Wang et al., 2019[51]). These findings clearly suggest that the lncRNA OTUD6B-AS1 can be considered as a promising target for RCC treatment.

## The Interplay between ncRNAs and Wnt/β-catenin Signaling in Bladder Cancer

Bladder cancer is the most common urological malignancy with the highest incidence and recurrence rates. It has been shown that more than 50 % of bladder cancer patients relapsed within the five years after their treatment resulting in unfavorable clinicopathological features and poor prognosis (Hussein et al., 2021[22]). Therefore, identifying novel therapeutic molecules is of great clinical importance for better management of bladder cancer. Recent investigation on the mechanism of action of lncRNAs indicates that some lncRNAs induce bladder cancer and metastasis by directly targeting certain tumor-suppressive miRNAs like miR-370-3p, miR-217, miR-214-3p, and miR-139-5p (Cheng et al., 2021[4]; Zhang et al., 2021[74]). Consistently, Zhang et al. discovered that lncRNA breast cancer anti-estrogen receptor 4 (BCAR4) was upregulated and associated with the more aggressive phenotype of bladder cancer. To further support the tumor stimulatory effects of BCAR4, it has been shown that the overexpression of this lncRNA induces bladder cancer proliferation and progression by inhibiting miR-370-3p and downregulating canonical Wnt axis in bladder cancer cells and tissues (Zhang et al., 2020[72]). 

Similarly, the expression of tumor-suppressive miR-217 was reported to be downregulated by lncRNA LINC01614 in bladder cancer. Increased expression of LINC01614 correlated with lower survival and poor clinical features in bladder cancer patients. Wang et al. reported that silencing LINC01614 inhibits tumor cell growth and proliferation through modulating miR-217 and reducing the Wnt signaling-related proteins including β-catenin and c-myc (Wang et al., 2021[54]). 

LncRNA X-inactive specific transcript (XIST) is another oncogenic lncRNA that induces bladder tumorigenesis through regulating miR-139-5p. Upregulation of lncRNA XIST was shown to be correlated with tumor aggressive features and metastasis in various malignancies including glioma, prostate, and bladder cancers (Liu et al., 2020[29]; Wang et al., 2021[52]). To evaluate the regulatory role of XIST on bladder tumorigenesis, Hu et al. illustrated that lncRNA XIST induces tumor growth and progression by suppressing miR-139-5p and finally inducing their downstream targets including Wnt1, β-catenin, and cyclinD1 proteins (Hu et al., 2017[18]).

There are multiple lncRNAs that induce cancer development and drug resistance by sponging certain tumor-suppressive miRNAs and preventing their regulatory effects on downstream target genes. For example, lncRNA nuclear-enriched abundant transcript 1 (NEAT1) accelerates tumor cell growth and induces Doxorubicin resistance in bladder cancer by targeting miR-214-3p (Guo et al., 2018[14]). The expression level of miR-214-3p has been reported to be closely associated with chemotherapy resistance in multiple tumors including breast, ovarian, and cervical cancers (Qin et al., 2019[36]; Yang et al., 2019[65]). Consistently, Guo et al. reported that the tumor suppressor miR-214-3p inhibits Wnt signaling through upregulation of GSK3β and AXIN2 and decreasing nuclear β-catenin protein levels. Further results indicate that the oncogenic effect of lncRNA NEAT1 is mediated by downregulation of miR-214-3p and activating canonical Wnt pathway in bladder cancer (Guo et al., 2018[14]). To further explore the role of lncRNAs in promoting chemoresistance, Xie et al. demonstrated the expression of lncRNA CDKN2B antisense RNA 1 (CDKN2B-AS) is positively related to a high pathological grade and decreased sensitivity to Gemcitabine through inducing Wnt/β-catenin signaling in bladder cancer (Xie et al., 2018[59]). 

As the Wnt signaling downstream genes are implicated in various cancer-related biological processes, it has been shown that some Wnt signaling regulatory ncRNAs are involved in tumorigenesis through regulating EMT and metastasis (O'Brien et al., 2020[32]; Zhang et al., 2021[70]). EMT enables tumor cells for metastasis through alleviating epithelial properties and developing mesenchymal phenotype. To further support the regulatory effect of Wnt signaling regulatory lncRNAs on the EMT process, the lncRNA DLX6 Antisense RNA 1 (DLX6-AS1), LINC00152, lncRNA long stress-induced non-coding transcript 5 (LSINCT5), and lncRNA cancer-associated region long non-coding RNA-7 (CARLO-7) were shown to be upregulated in bladder cancer and induce tumor growth, invasion, and metastasis by inducing Wnt signaling and modulating the expression of EMT-related proteins including vimentin, N-cadherin, and E-cadherin proteins (Zhu et al., 2018[79]; Guo et al., 2019[13]; Xian-Li et al., 2019[58]; Huang et al., 2020[20]).

In addition to the cancer-related lncRNAs mentioned above, there are several tumor-suppressive lncRNAs that inhibit bladder tumorigenesis through down-regulating canonical Wnt pathway. For instance, the lncRNA miR143HG was shown to be suppressed in bladder cancer which is negatively associated with tumor growth and development. Mechanistically, lncRNA miR143HG functions as a ceRNA for miR-1275 to upregulate AXIN2. Ectopic expression of miR143HG inhibits cell cycle progression and alleviates tumor cell proliferation by suppressing Wnt/β-catenin signaling (Xie et al., 2019[60]). Moreover, downregulation of lncRNA cancer susceptibility candidate 2 (CASC2) was reported in various tumors including glioma, endometrial, and RCC which is correlated with tumor malignant features and worse prognosis (Ghafouri-Fard et al., 2020[12]). Pei et al. reported that enforced expression of CASC2 reduces cell proliferation and metastasis through inactivating Wnt pathway and promoting apoptosis in bladder cancer (Pei et al., 2017[33]).

## The Interplay between ncRNAs and Wnt/β-catenin Signaling in Prostate Cancer

Prostate cancer is the second cause of cancer-related death in males worldwide and its incidence is elevating every year. Despite recent advances in therapeutic methods, the overall survival of patients with prostate cancer still remains low (Sun, 2021[49]). To investigate the novel therapeutic targets for prostate cancer therapy, several lncRNAs are identified that induce prostate tumorigenesis, metastasis, and chemoresistance by targeting certain tumor-suppressive miRNAs including miR-29a-3p, miR-212-5p, miR-30a-5p, miR-324-3p, and miR-452-5p. 

The lncRNA bladder cancer-associated transcript 1 (BLACAT1) is shown to be upregulated in various types of human carcinomas including endometrial, NSCLC, osteosarcoma, cervical, and prostate cancers (Liao et al., 2021[28]). Elevated expression of lncRNA BLACAT1 is correlated with pro-malignant features of cancer and lower survival time in patients with prostate cancer. In support of the oncogenic mechanism of BLACAT1 on prostate cancer, Liao et al. demonstrated that this lncRNA induces tumor cell growth and proliferation by targeting miR-29a-3p and inducing Wnt signaling modulatory proteins like disheveled segment polarity protein 3 (DVL3). Upregulation of DVL3 was reported in various human cancers which was related to tumor growth and recurrence rate by promoting the Wnt/β-catenin axis (Liao et al., 2021[28]).

LINC00115 is another onco-lncRNA that promotes prostate tumorigenesis and metastasis through targeting miR-212-5p. Tumor suppressor miR-212-5p was shown to inhibit tumor cell proliferation through repressing the expression of Frizzled Family Receptor 5 (FZD5). Increased expression of FZD5 was shown to be implicated in cancer development and metastasis by over-activation of canonical Wnt pathways in various cancers including HCC and prostate cancers. Therefore, the LINC00115/miR-212-5p/FZD5 axis can be regarded as a promising target for treatment of prostate cancer (Peng et al., 2021[34]). 

Moreover, the lncRNA non-coding RNA activated by DNA damage (NORAD) was shown to be dysregulated in various tumors including lung, osteosarcoma, ovarian, and prostate cancers (Soghli et al., 2021[46]). Enhanced expression of NORAD induces tumor cell proliferation, EMT, and metastasis through downregulating miR-30a-5p (Zhang and Li, 2020[76]). The tumor suppressor miR-30a-5p was shown to inhibit cell proliferation, migration, and tumor growth in various human malignancies such as CRC, NSCLC, breast, and gallbladder cancers (Jiang et al., 2018[24]). In agreement with these findings, Zhang and Li reported that the oncogenic activity of lncRNA NORAD in prostate carcinogenesis can be mediated by sponging miR-30a-5p and modulating Wnt signaling and EMT-related proteins including vimentin, snail, N-cadherin, and E-cadherin (Zhang and Li, 2020[76]).

In addition to NORAD, the small nucleolar RNA host gene 7 (SNHG7) and lncRNA SOX2 overlapping transcript (SOX2-OT) are identified as EMT-related lncRNAs that are involved in prostate cancer progression and metastasis. The oncogenic activity of SNHG7 and SOX2-OT are shown to be mediated via targeting miR-324-3p and miR-452-5p respectively. It has been shown that downregulation of these tumor-suppressive miRNAs by SNHG7 and SOX2-OT induces the canonical Wnt route and promotes malignant features in prostate cancer cells by modulating EMT-related proteins (Hu, 2019[17]; Song et al., 2020[48]). Taken together, these findings revealed that the EMT-related lncRNAs may be considered as promising targets for prostate cancer therapy.

## Conclusion

**Figure 1:**
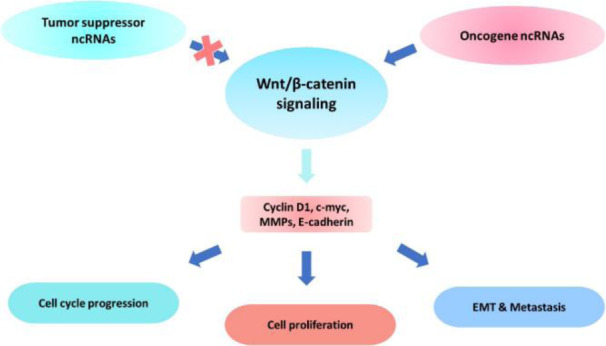
Schematic representation of regulatory effects of ncRNAs on the activity of the Wnt/β-catenin signaling contributed to the pathogenesis of UT cancers

**Figure 2:**
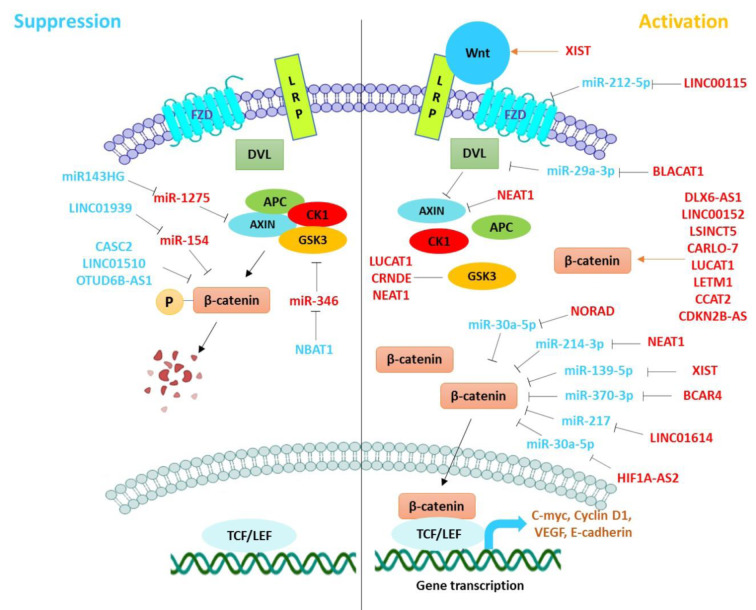
The interplay between lncRNAs and miRNAs in regulating Wnt/β-catenin signaling pathway

In this study, we summarized the recent findings on the tumor-suppressive or oncogenic function of lncRNAs and miRNAs in modulating canonical Wnt signaling that is involved in the pathogenesis of UT cancers (Figure 1(Fig. 1)). LncRNAs and miRNAs exert crucial roles in tumor growth, invasion, and metastasis through regulating the expression of various genes and proteins at transcriptional and post-transcriptional levels (Tables 1(Tab. 1) and 2(Tab. 2)). Emerging evidence highlights the potential of these ncRNAs in diagnostic and therapeutic uses. Considering the complex interplay between lncRNAs and miRNAs in UT cancers (Figure 2(Fig. 2)), it has been suggested that downregulation of oncogenic, or restoration of tumor suppressor ncRNAs may repress the malignant features of UT cancers. Taken together, further studies on cross-talk between lncRNAs and miRNAs in regulating the Wnt/β-catenin signaling will provide novel therapeutic targets for the treatment of UT cancers.

## Declaration

### Ethical approval

This article does not contain any studies with human participants performed by any of the authors.

### Conflict of interest

The authors declare no conflict of interest in this article.

### Funding

This research was partly supported by Mashhad University of Medical Sciences (Farzad Rahmani).

## Figures and Tables

**Table 1:**
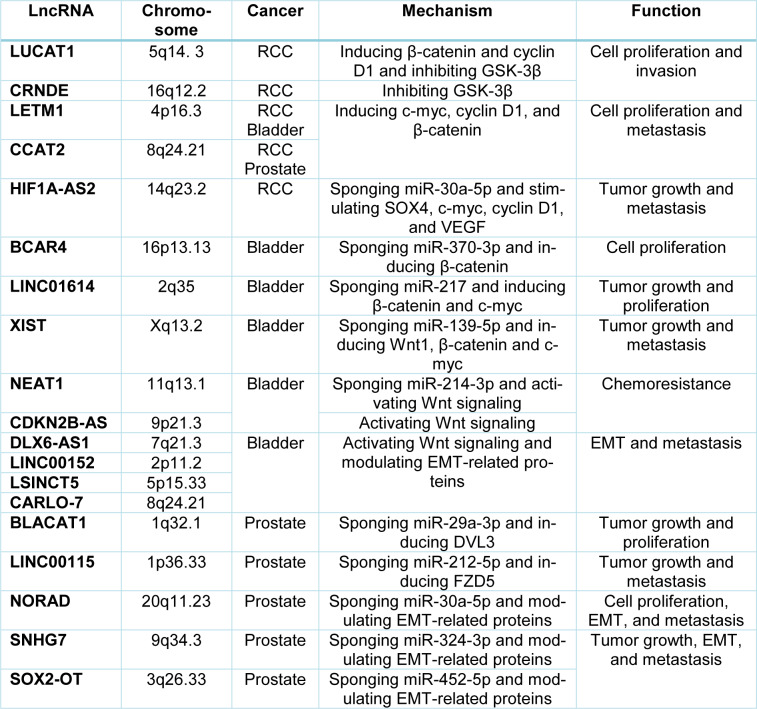
Oncogenic lncRNAs

**Table 2:**
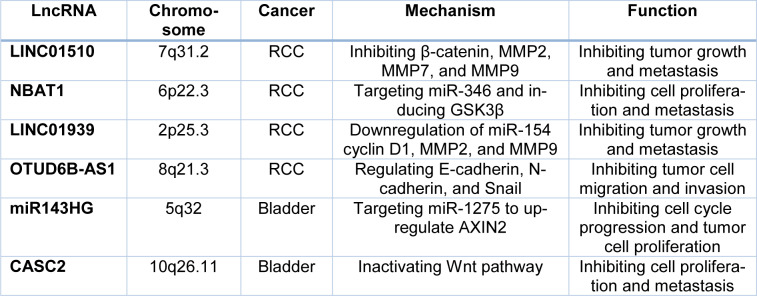
Tumor suppressive ncRNAs
